# Repurposing doxycycline for Alzheimer's treatment: Challenges from a nano-based drug delivery perspective

**DOI:** 10.1016/j.bbih.2024.100894

**Published:** 2024-10-25

**Authors:** Mariana Conceição, Leonardo Delello Di Filippo, Jonatas Lobato Duarte, Fernando Pereira Beserra, Maria Palmira Daflon Gremião, Marlus Chorilli

**Affiliations:** a Department of Drugs and Medicines, School of Pharmaceutical Sciences, São Paulo State University (UNESP), Araraquara, São Paulo, Brazil; b Institute of Biosciences, São Paulo State University (UNESP), Botucatu, São Paulo, Brazil; c School of Pharmaceutical Sciences of Ribeirão Preto, University of São Paulo (USP), Ribeirão Preto, São Paulo, Brazil

**Keywords:** Doxycycline, Alzheimer, Nanotechnology, Drug repurposing, Drug repositioning, Drug delivery

## Abstract

Drug repurposing, also known as drug repositioning, involves identifying new applications for drugs whose effects in a disease are already established. Doxycycline, a broad-spectrum antibiotic belonging to the tetracycline class, has demonstrated potential activity against neurodegenerative diseases like Alzheimer's and Parkinson's. However, despite its promise, the repurposed use of doxycycline encounters challenges in reaching the brain in adequate concentrations to exert its effects. To address this issue, nanostructured systems offer an innovative approach that can enhance brain targeting and the desired therapeutic outcomes. This review discusses the advances in doxycycline repurposing for Alzheimer's disease, presenting physicochemical and biological aspects that permeate doxycycline's repositioning and its application in nano-based delivery systems.

## Highlights

• Doxycycline has shown potential activity against beta amyloid-induced memory impairment;
• Doxycycline's physicochemical properties allows its encapsulation by nanoparticles;
• Doxycycline applied into nano-based delivery systems may improve brain targeting.

## Graphical abstract

**Figure:**
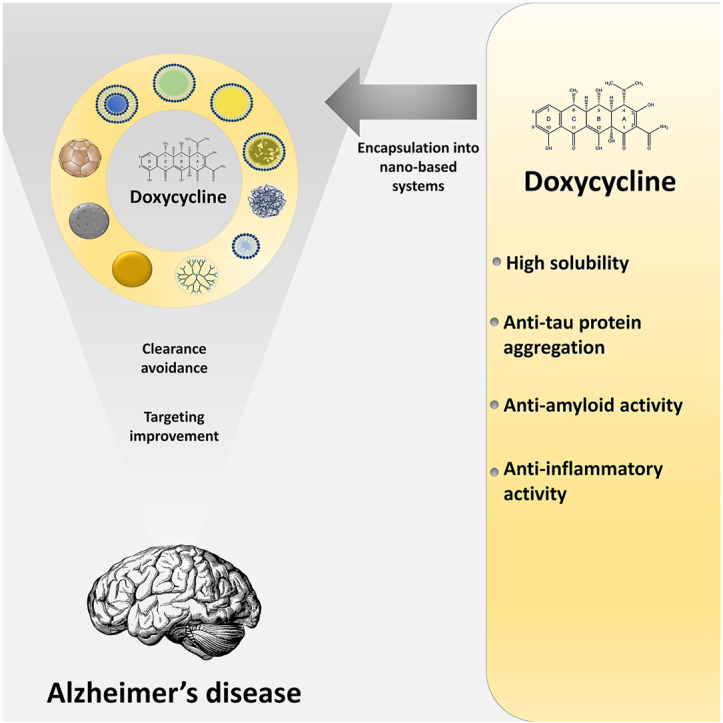

## 1 Introduction

Drug discovery demands significant time and financial resources, often falling short of the urgent need for disease treatment or cure (Parvathaneni et al., 2019). Developing and distributing a new drug typically takes 10–15 years, with a high clinical failure rate (around 90%) and requiring an estimated investment of $0.8–1.5 billion dollars (Cai et al., 2020; DiMasi et al., 2016). To overcome this challenge, drug repurposing has emerged as a strategy, utilizing clinically approved drugs for new therapeutic indications, thus reducing time and costs (Jourdan et al., 2020).

Doxycycline (DOX) is a second-generation tetracycline antibiotic widely used to treat gastrointestinal and respiratory infections, malaria, acne, rosacea, anthrax, and as a food additive to enhance livestock growth (Henehan et al., 2017; Holmes and Charles, 2009; Kogawa and Salgado, 2012). Recently, research has explored its potential as a repurposed drug to treat other diseases such as COVID-19 (Malek et al., 2020), dengue (Fredeking et al., 2015), some types of cancer (Ali et al., 2018), Alzheimer's, and Parkinson's diseases (Dominguez-Meijide et al., 2021).

Neurodegenerative diseases, such as Alzheimer's and Parkinson's, could significantly benefit from drug repurposing, given the lack of effective treatments and the absence of a cure. These conditions involve toxic protein aggregates such as tau fibrils and beta-amyloid (Aβ) plaques, which drive degeneration and cause symptoms like memory loss and cognitive decline (Alzheimer's Association, 2022; Zaki et al., 2022; Fonseca-Santos et al., 2023; Nascimento et al., 2022; Ribeiro et al., 2022). Drugs capable of interacting with and neutralizing these proteins, such as DOX, nilvadipine, and rifampicin, offer promise (Dominguez-Meijide et al., 2021; Socias et al., 2018). However, challenges persist, including efficient brain delivery and targeting to avoid interactions with other organs. Incorporating drugs into nanoparticles presents a viable solution to these limitations.

Nanoparticles, being structures of reduced size (ranging from 1 to 100 nm), offer numerous advantages. They boast a larger surface area at the site of action, are easily absorbed by cells, and have the capacity to carry larger amounts of drug (Ghasemiyeh and Mohammadi-Samani, 2018; Hulla et al., 2015). Consequently, the use of nanoparticles as drug carriers is associated with enhanced bioavailability, solubility, stability, dissolution rates, and biocompatibility (Bhatia, 2016). Moreover, by functionalizing the nanoparticle surface with molecules that have an affinity for the target tissue, drug delivery can be further improved (Cagliani et al., 2019).

Considering DOX's repurposing potential and the benefits of a nanotechnological approach to Alzheimer's disease (AD) treatment, understanding the drug's molecular and chemical characteristics, as well as its interactions in AD pathophysiology, is crucial. Therefore, this review explores repurposing DOX for AD treatment, focusing on its physicochemical properties for encapsulation in nanostructured systems. The review results from an extensive search for English-language articles across various databases, including PubMed, ScienceDirect, Google Scholar, Cochrane Database of Systematic Reviews, Scopus, LILACS, and Medline.

## 2 Doxycycline physicochemical properties

Understanding the physicochemical properties of repurposed drugs is crucial for optimizing their absorption, distribution, and efficacy, as outlined in their pharmacokinetics and pharmacodynamics. These properties also impact drug encapsulation and release efficiency in delivery systems (Zhao et al., 2019). The following paragraphs discuss the key properties of DOX and their implications for innovative formulation development.

DOX (CAS number: 24390-14-5) is an odorless yellow crystalline powder with a bitter taste, characteristic of antibiotics in the tetracycline drug class (Nelson, 1998). Its yellow color results from a phenolic group in its chemical structure, which absorbs light in the visible spectrum. DOX is soluble in water, making it suitable for oral administration, but its solubility varies with pH. It is less soluble in neutral and alkaline solutions, potentially limiting its absorption and bioavailability in the body (Bogardus and Blackwood, 1979).

DOX is classified under the Biopharmaceutics Classification System (BCS) as class I (BCS-I), indicating high solubility and permeability (Charalabidis et al., 2019). This classification also designates DOX as a biowaiver drug, meaning that *in vitro* results on drug quality suffice to predict *in vivo* pharmacokinetic properties (Abraham et al., 2021; Jantratid et al., 2010). The chemical formula of DOX is C_22_H_24_N_2_O_8_, resulting in a molecular weight of approximately 444.44 g/mol, which is relatively high for a small molecule drug. The high molecular weight of DOX can restrict its diffusion through cell membranes and its excretion by the kidneys. However, it can also confer desirable properties such as a high volume of distribution and a long biological half-life (Jantratid et al., 2010).

Molecular weight and solubility are critical factors affecting a molecule's ability to penetrate the blood-brain barrier for Alzheimer's disease (AD) treatment (Bickel, 2005). Central nervous system (CNS) drugs typically possess low molecular weight, few polar surfaces, and a LogP (partition coefficient) between 1.5 and 2.5 (Pajouhesh and Lenz, 2005; Singh et al., 2020). To mitigate alterations in drug bioavailability caused by hepatic metabolism and passage through the blood-brain barrier (BBB), intranasal administration offers a promising alternative, as it bypasses the BBB (Bickel, 2005; Mistry et al., 2009). Additionally, incorporating DOX into a nano-based drug delivery system may address solubility issues (Thangudu et al., 2020).

**Fig. 1:**
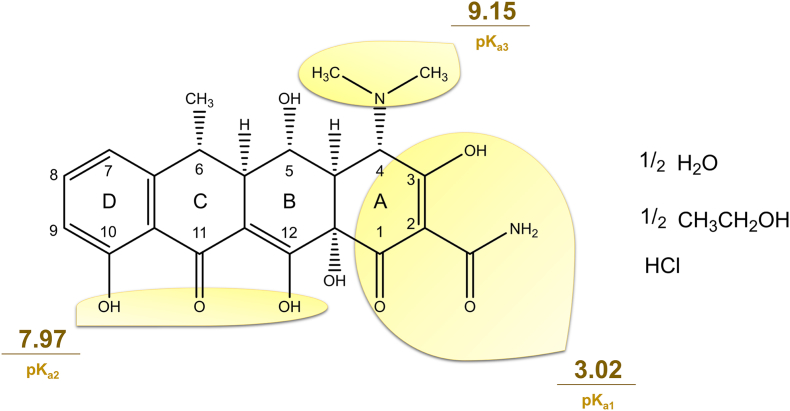
Chemical structure of doxycycline hyclate with pKa values for each main functional group. pK_a1_: tricarbonyl system; pK_a2_: phenolic diketone system; pK_a3_: dimethylammonium. CAS number 24390-14-5.

Doxycycline hyclate (Fig. 1) is the most commonly used salt form of DOX in pharmaceutical formulations, although it can be converted into other forms with distinct physicochemical properties (Kogawa and Salgado, 2012). It has a molecular weight of 512.94 g/mol and a melting point of 201 °C. DOX hyclate can be dissolved in water and neutralized with sodium hydroxide to obtain doxycycline monohydrate (DOX.H_2_O). Moreover, adding hydrochloric acid to this form yields doxycycline hydrochloride (DOX.HCl.H_2_O) (Kogawa and Salgado, 2012). DOX hyclate exhibits high water solubility, reaching 50 mg/mL at pH 2.16, and demonstrates hydrophilic antimicrobial properties with a LogP value of −0.25 (Bogardus and Blackwood, 1979). This drug form is considered highly soluble and permeable, as confirmed by crystallographic analyses (Jantratid et al., 2010; Legendre et al., 2012).

The pKa values of DOX are reported as 3.02 (pKa1), 7.97 (pKa2), and 9.15 (pKa3), indicating the presence of three ionizable groups. The first pKa corresponds to the dissociation of the tricarbonyl system at ring A (Carbons 1–3), the second to the ketophenolic system (C10 and C12), and the third to the dimethylamino group (C4), as depicted in Fig. 1 (Kogawa and Salgado, 2012; Bolobajev et al., 2016; Guerra et al., 2006; Shariati et al., 2009).

The ionization of DOX influences its solubility and absorption in the body. In acidic environments, like the stomach, DOX becomes more ionized and hence more soluble. Conversely, in alkaline environments such as the blood and tissues, DOX is less ionized and therefore less soluble (Bogardus and Blackwood, 1979; Shariati et al., 2009).

As previously mentioned, DOX's phenolic group (Fig. 1) absorbs light in the visible spectrum, rendering DOX highly photosensitive. Exposure to light can alter not only the drug's molecular structure but also its storage quality and biological effects in formulations (Kogawa et al., 2014; Pandey et al., 2022). To enhance DOX's photostability, options include creating inclusion complexes of the drug with macromolecules like cyclodextrin, adding excipients such as antioxidants or coating agents, or even incorporating the drug into nanoparticles, as discussed in the review by Janga et al., 2018) (Janga et al., 2018).

Analytical techniques such as spectrophotometry, X-ray diffraction, thin-layer chromatography, and high-performance liquid chromatography (HPLC) are employed to detect molecular modifications and verify the purity of DOX. These methods are also applied to various matrices (plasma, seminal fluid, milk, tissues, soil, water, etc.) due to DOX's use in microbial infection treatment, animal growth promotion, and environmental monitoring in water bodies and soil (Kogawa and Salgado, 2012; Ghaemi and Absalan, 2015; Xu et al., 2021). *In silico* or computational models offer another effective approach for understanding potential interactions between DOX and organic or inorganic molecules. They provide insights into the mechanisms of action in various organs (Kwon et al., 2019).

Therefore, DOX's good water solubility, desirable for most administration routes and organs, requires modifications for brain targeting to optimize its action. These modifications may include encapsulation or altering its administration route, both of which will be discussed in the following sections.

## 3 Doxycycline as an Alzheimer's disease therapeutic agent

**Fig. 2:**
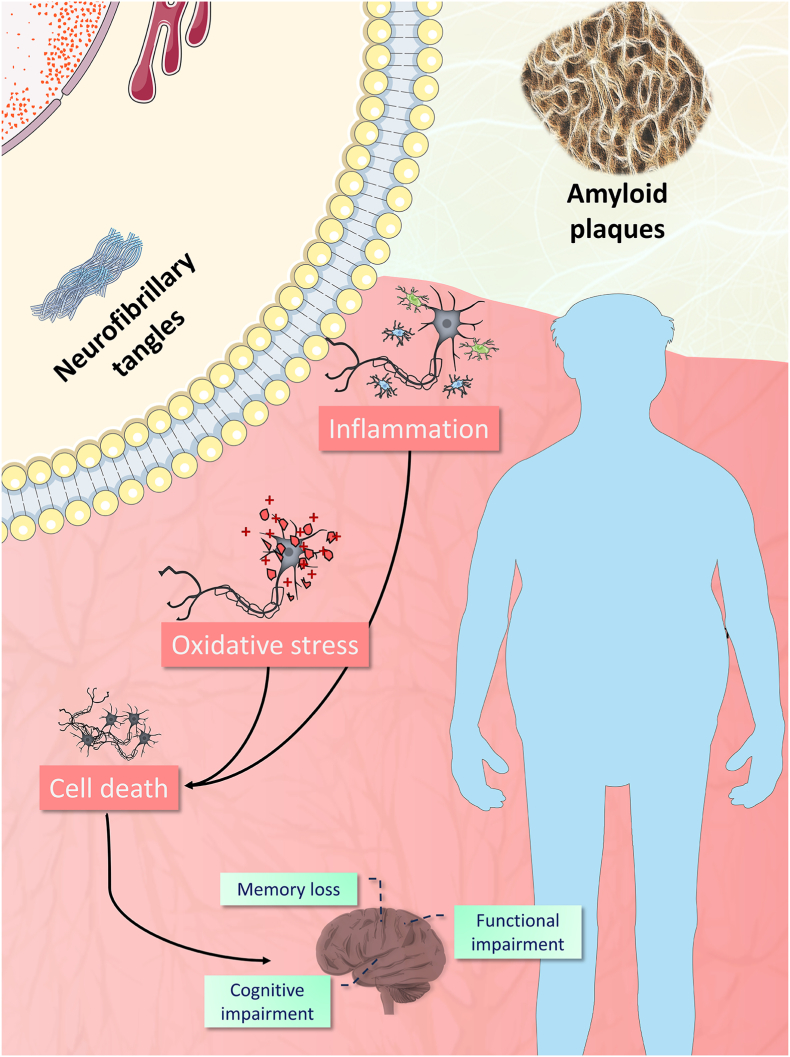
Schematic representation of Alzheimer's disease according to the amyloid and tau hypothesis. (A) Aβ proteins aggregated in amyloid plaques accumulate in the extracellular environment leading to inflammation, oxidative stress, and cellular death; (B) tau proteins accumulate as intracellular neurofibrillary tangles triggering inflammation, oxidative stress that also culminates in cellular death. The Figure was partly generated using Servier Medical Art, provided by Servier, licensed under a Creative Commons Attribution 3.0 unported license.

Alzheimer's disease, despite its increasing global prevalence, lacks a cure or effective treatment strategies (World Alzheimer Report, 2022). Symptoms like memory loss, cognitive decline, and functional impairment are linked to neuron death, particularly in the hippocampus (Huang et al., 2020). ccording to widely accepted pathogenesis theories of Alzheimer's, such as the amyloid and tau hypothesis, neurodegeneration is driven by oxidative and inflammatory processes associated with the accumulation of toxic proteins, primarily Aβ and hyperphosphorylated tau proteins. Aggregated, these proteins form neurofibrillary tangles and amyloid plaques, respectively (Fig. 2) (Calabrò et al., 2021).

Current Alzheimer's disease (AD) treatment includes drugs like donepezil, galantamine, and rivastigmine, known as cholinesterase enzyme inhibitors. These drugs delay symptom progression by sustaining acetylcholine levels and preserving cholinergic synapses, crucial for cognitive functions (Chen et al., 2022; Long and Holtzman, 2019). None of these strategies, however, act towards the main cause of brain degeneration. Since then, more disease specific and effective treatments are in search.

Aβ proteins result from the cleavage of Amyloid precursor protein (APP) by secretase enzymes. This process yields peptides of varying lengths, typically ranging from 38 to 43 amino acids. Aβ42 and Aβ40 forms – named based on the number of amino acids composing them following APP cleavage - are predominantly implicated in the pathogenesis of AD due to their heightened tendency to aggregate in the extracellular matrix (Hefter et al., 2020). Aβ42, in particular is more fibrillogenic than Aβ40 and leads to the formation of neurotoxic aggregates (Kuperstein et al., 2010). Once aggregated into fibrils, Aβ becomes highly stable, exhibiting insolubility and resistance to denaturation induced by factors like heat, sonication, pH variations, and certain organic solvents (Sahoo et al., 2020). In this context, Aβ deposits initiate inflammatory and oxidative responses within neurons, initiating a cascade of events culminating in cellular death, as depicted in Fig. 2 (Mehta and Mehta, 2023).

Based on this hypothesis, Forloni et al. (2001) (Forloni et al., 2001) investigated the anti-amyloidogenic activity of tetracycline and DOX by analyzing their binding to synthetic Aβ42 proteins using the Thioflavin T (ThT) assay. They also examined whether the drugs altered the protease digestion resistance of amyloid aggregates. The results revealed reduced quantities of amyloid aggregates and increased enzymatic degradation when Aβ42 peptides were co-incubated with DOX or tetracycline for 5 days. Additionally, Forloni and colleagues reported that tetracyclines exhibited defibrillogenic action against pre-formed amyloid fibrils (Forloni et al., 2001).

Forloni's group confirmed the binding affinity of tetracyclines to amyloid proteins, a finding supported by a computational model of molecular dynamics conducted by Gautieri et al. (2019) (Gautieri et al., 2019). Their simulation revealed that DOX binds to exposed hydrophobic amino acid residues, destabilizing fibrils through non-polar interactions. This binding and destabilization mechanism prevents the formation of protein oligomers, which are considered more toxic to neuronal cells (Gautieri et al., 2019).

The idea of DOX as a potential treatment for AD originated from studies that initially utilized oral administration for drug delivery (Gianni et al., 1995). Subsequent to these studies, two clinical trials with oral DOX for AD treatment were conducted, one in 2004 (Loeb et al., 2004) and another in 2013 (Molloy et al., 2013), providing significant insights into this area of research. In the first trial, Loeb et al. (2004) investigated the effects of two antibiotics on AD treatment, guided by the amyloid and infectious AD hypothesis. According to this infectious hypothesis, infections caused by viruses, prions, or bacteria contribute to the development of a chronic inflammatory environment in the brain, ultimately resulting in cell death (Seaks and Wilcock, 2020). Loeb's group suggested that *Chlamydia pneumoniae*, a gram-negative bacteria causing respiratory infections, could be involved in AD etiology by generating inflammatory mediators (Balin et al., 1998; Shima et al., 2010). Both antibiotics were capable of penetrating the blood-brain barrier and interacting with amyloid proteins, potentially disrupting aggregates and reducing cognitive impairment. Therefore, mild to moderate AD patients received daily oral doses of DOX (200 mg) and rifampin (300 mg) or placebo for three months, and their cognitive symptoms were assessed for twelve months using the Standardized Alzheimer's Disease Assessment Scale (SADAS) cognitive subscale. Additionally, serum *C. pneumoniae* antibody detection was performed. The results indicated that DOX and rifampin treatment led to reduced dysfunctional behavior after six months according to the SADAS cognitive subscale, attributed to the drugs' ability to impair amyloid aggregate formation. However, this effect was not sustained after twelve months. Furthermore, antibiotic treatment did not affect *C. pneumoniae* antibody levels in serum (Loeb et al., 2004).

In 2013, the DARAD (Doxycycline and Rifampin for Alzheimer's Disease) trial, a continuation of Loeb et al., 's 2004 study, was conducted by the Molloy group. This larger clinical trial analyzed the cognitive, global function, and behavioral aspects of AD patients over one year. A total of 4006 patients received oral DOX 100 mg twice a day and rifampin 300 mg daily or DOX 100 mg twice a day with placebo daily, and placebo 100 mg twice a day and rifampin 300 mg daily during the 12 months. The therapeutic hypothesis focused on the anti-amyloid, anti-inflammatory, and anti-tau activities of DOX and rifampin. Despite their potential, the trial was halted prematurely at the request of the Data Monitoring Committee due to the treatments not demonstrating superiority over the placebo. Consequently, it was concluded that neither oral rifampin nor DOX provided benefit against AD (Molloy et al., 2013).

While clinical trials have not yielded strong results regarding DOX's efficacy in AD, they have provided valuable insights into the drug's effects through oral administration, which will be discussed further. Additionally, research using experimental models continues, exploring various disease etiology theories such as the tauopathy hypothesis.

According to the tauopathy hypothesis, aberrant hyperphosphorylated tau proteins accumulate intracellularly, triggering inflammatory and oxidative stress conditions (Mehta and Mehta, 2023). Tau proteins are physiologically responsible for the rearrangement and stabilization of microtubules within the cell, being classified as microtubule-associated proteins. However, in pathological conditions, tau proteins aggregate to form insoluble intracellular filaments called neurofibrillary tangles, disrupting molecular and organelle trafficking and leading to inflammation and cell death (Brandt et al., 2020; Sinsky et al., 2021). The search for anti-tau drugs closely follows the search for anti-Aβ protein drugs due to the importance given to these proteins in the etiology of AD. Next, we will discuss the impact of DOX on the aggregation of tau proteins.

Medina et al. (2021) (Medina et al., 2021) investigated the influence of DOX on the aggregation of monomeric tau proteins using an *in vitro* heparin-induced fibrillization model. They found that DOX inhibited tau aggregation in a dose-dependent manner, as evidenced by ThT and Congo Red binding analysis. Additionally, DOX altered the kinetics of tau aggregation by both inhibiting and disrupting aggregates, as observed through a Bis-ANS fluorescent probe. Importantly, this probe indicated that DOX treatment resulted in reduced exposure of tau hydrophobic residues compared to control samples. Consequently, the action of the drug led to a structurally modified version of tau protein with reduced aggregation propensity (Medina et al., 2021). While this study elucidated many of the mechanistic interactions between DOX and tau proteins in the context of AD through *in vitro* studies, *in vivo* research has also played a significant role in understanding the disease's pathogenesis.

Balducci et al. (2018) (Balducci et al., 2018) investigated the effects of DOX in different models: transgenic APP/PS1 mice expressing abundant APP and mutant presenilin 1, and C57BL/6 mice with memory impairment induced by intracerebroventricular (i.c.v.) injection of synthetic Aβ42 oligomers (AβOs) in an acute model of AD. AβOs are intermediate Aβ oligomeric forms that are produced during fibrillization also known as Aβ-derived diffusible ligands (ADDLs), which are soluble but toxic variants (Verma et al., 2015).

In the study, TgAPP/PS1dE9 transgenic mice, which express a mutant human presenilin 1 received chronic (20 or 60 days) and acute doxycycline (2 h) treatments of intraperitoneal (i.p.) DOX diluted in saline at 10 mg/kg/mL. The control group received saline only. Transgenic mice receiving i.p. doxycycline for 20 days showed improved memory without a significant difference in the number of amyloid plaques compared to the wild-type group. Similar results were observed in groups treated with doxycycline for 60 days. When assessing the presence of ABOs in brain extracts, no significant decrease in ABO species was observed after either treatment period. They also tested whether acute i.p. doxycycline treatment, administered 2 h before the Novel Object Recognition Task test, would improve memory. Once again, doxycycline improved memory. They concluded that doxycycline might enhance memory by counteracting the actions of ABOs rather than reducing their levels.

For the C57BL/6 mice, acute memory impairment was induced via intracerebroventricular (i.c.v.) injection of synthetic Aβ42 oligomers (AβOs). Two treatments were tested: doxycycline at 30 μM or 70 μM co-incubated with AβOs 1h before i.c.v. injection, and DOX at 25, 50, and 100 mg/kg administered i.p. 2h before AβOs i.c.v. injection. The control groups received either AβOs or saline only. Acute i.p. DOX treatment administered 2h before the Novel Object Recognition Task test improved memory, suggesting that DOX might counteract AβOs actions rather than reduce their levels. The acute memory impairment model in C57BL/6 mice, created by Balducci et al. (2010), was used to test doxycycline's ability to antagonize ABOs as a memory impairment factor (Balducci et al., 2010). Pre-incubating doxycycline at 30 and 70 μM with ABOs before i.c.v. injection did not prevent memory impairment and i.c.v. injection of doxycycline alone had a harmful effect on memory. Therefore, they tested i.p. doxycycline at three concentrations (25, 50, and 100 mg/kg) administered 2 h before ABOs i.c.v. injection. This approach showed that doxycycline counteracted ABOs actions in a dose-dependent manner (Balducci et al., 2018).

To enhance translational validity, reduce animal usage, and minimize costs, researchers have turned to alternative *in vivo* models like zebrafish and *Drosophila* to simulate neurodegenerative diseases (Mullane and Williams, 2019). Some studies have utilized these models to investigate potential treatments for AD, including DOX. By using an alternative AD model of transgenic *Drosophila melanogaster* expressing high amounts of Aβ peptides and related neurodegeneration features, Costa et al. (2011) (Costa et al., 2011) tested whether DOX added to fly food would improve behavior, longevity, and Aβ metabolism. QUANTO TEMPO RECEBERAMWhile DOX didn't affect Aβ expression or fly longevity, it did improve locomotion and climbing index in Aβ42-expressing flies in a dose-dependent manner. Additionally, *in vitro* tests demonstrated DOX's anti-aggregation effects against Aβ42 proteins, reducing aggregate size and producing thinner and shorter fibrils compared to samples without the drug as assessed by ThT assay (Costa et al., 2011). Costa and colleagues investigated caspase-3 activation, a pathway that leads to apoptosis, after treating an SH-SY5Y (neuroblastoma) cell culture with Aβ42. They found that Aβ42 induced apoptosis in the cells through caspase-3 activation. However, when DOX was introduced during the Aβ42 aggregation phase, apoptosis decreased, confirming the drug's effectiveness against Aβ42 toxicity (Costa et al., 2011). These results aid the assumptions that DOX delayed disease progression and could be a valuable resource to prevent Aβ42 aggregation and toxicity as the more insoluble and prone to aggregation as a variant of amyloid beta peptide involved in AD (Kuperstein et al., 2010).

The evidence-based benefits of DOX are numerous towards AD's treatment, mostly based on its anti-aggregating activity. A range of experimental models and even clinical trials were carried out aiming at the successful results that were predicted by computational and *in vitro* assays. DOX's safety profile after oral use is established even in long term conditions as shown by Waitayangkoon et al. (2023) in a systematic review of clinical trials that analyzed doxycycline and other antibiotics use for more than 6 months. The results revealed no severe adverse events. Commonly reported tolerable reactions in the 21 analyzed clinical trials included gastrointestinal disturbances, photosensitivity, and rash (Waitayangkoon et al., 2023). Nevertheless, not all results occurred as expected. The following section explores nanotechnology to enhance DOX's efficacy against AD.

## 4 Nanotechnology and nano based drug delivery systems in health: concepts and examples

**Fig. 3:**
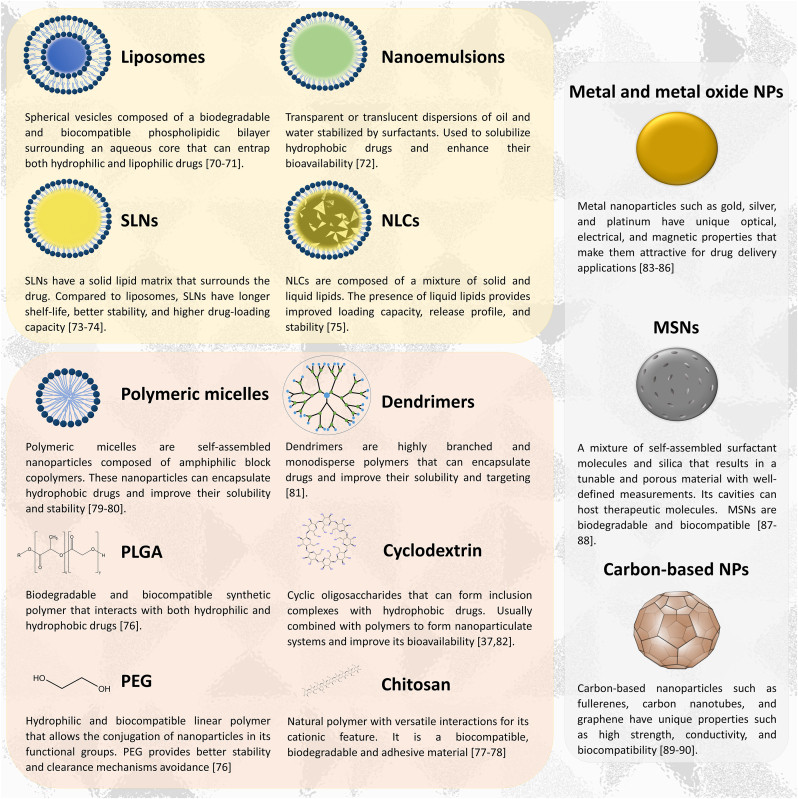
Different types of nanoparticles with brief description. Liposomes, nanoemulsions, SLNs, and NLCs are classified as lipid nanoparticles. Polymeric nanoparticles are represented by polymeric micelles, dendrimers, PLGA, cyclodextrin, PEG and chitosan. PEG and PLGA are polymeric molecules used in the surface of nanoparticles with distinct materials, not only polymeric, to improve stability and targeting. Metal, metal oxide, carbon-based nanoparticles and MSNs are in the group of inorganic nanoparticles while the other ones are organic. MSNs = mesoporous silica nanoparticles; NLCs = nanostructured lipid carriers; SLNs = solid lipid nanoparticles; PEG = polyethylene glycol; PLGA = poly(lactic-co-glycolic acid. The Figure was partly generated using Servier Medical Art, provided by Servier, licensed under a Creative Commons Attribution 3.0 unported license.

Nanotechnology involves designing, producing, and manipulating materials at the nanoscale, where “nano” represents the billionth part of a meter or the size of individual atoms and molecules. This precise control enables scientists and engineers to develop materials and devices with unique properties and applications compared to bulk materials. (Bayda et al., 2019). Nanotechnology holds vast potential in healthcare, particularly in developing novel materials and devices that interact with biological systems. For instance, nanoparticles can deliver drugs directly to cancer cells, enhancing treatment efficacy with fewer side effects. With sizes ranging from 1 to 100 nm, nanoparticles possess unique physical and chemical properties, making them ideal for biomedical applications, including drug delivery for treating CNS diseases as ilustrated in Fig. 3 and Table 1 (Anjum et al., 2021).

**Table 1:** Doxycycline-related applications for different types of nanostructured systems as drug delivery systems.

Nanoparticle	Applications with doxycycline
Liposomes	Doxycycline and apigenin loaded liposomes inhibited photo-aging processes by reducing oxidative stress, inflammation, and metalloproteinases activation in *in vitro* and *in vivo* assays (Liu et al., 2023); Doxycycline loaded nanoliposomes presented improved bactericidal activity against *Staphylococcus epidermidis* and *Staphylococcus aureus* (Savadi et al., 2022).
Nano/microemulsions	Doxycycline nanoemulsions presented prolonged or similar post-antibiotic effects against *S. aureus* when compared to the non-encapsulated version of the drug (Mohamed et al., 2021).
SLNs	Levofloxacin and doxycycline encapsulated in solid lipid nanoparticles were efficiently delivered to the brain through the intranasal route as confirmed by *in vivo* studies (Abdel Hady et al., 2020); solid lipid nanoparticles efficiently encapsulated doxycycline and the formulation was more effective in the treatment of brucellosis than the free drug (Hosseini et al., 2019).
NLCs	NLCs containing a chemically modified tetracycline efficiently encapsulated the drug and provided its entry in HeLa cells (Yang et al., 2013).
PLGA	Doxycycline hydrochloride-loaded PLGA nanoparticles showed increased efficacy as antifilarial drug when administered subcutaneously when compared to the unencapsulated version (Singh et al., 2016).
PEG	
Chitosan	Doxycycline and atorvastatin loaded chitosan nanoparticles efficiently delivered the drugs with sustained release and antimicrobial activity against *Staphylococcus aureus* (Yıldırım et al., 2023); doxycycline hydrochloride loaded chitosan nanoparticles presented anti-psychotic activity with increased GABA an GSH levels and decreased TNF-α and dopamine levels in a psychosis mice model (Yadav et al., 2017).
Micelles	Doxycycline partitioning in a micelle system composed of hexadecyltrimethylammonium bromide alters the drug's interaction with the target protein, albumin (Tinku and Choudhary, 2023); pH influences the inclusion of doxycycline in ammonium surfactant micelles and Mg^+^ ions induces the drug's release from the system (Cesaretti et al., 2014)
Dendrimers	Dendrimer based-hydrogels were efficient in the controlled delivery of doxycycline hydrochloride in tissue adhesive (Wang et al., 2021).
Cyclodextrin	Hydroxypropyl-β-cyclodextrin inclusion increased doxycyclines's photostability (Kogawa et al., 2014); sulfobutylether-β-cyclodextrin increased doxycycline's thermal resistance (Carvalho et al., 2023).
Metal nanoparticles	Doxycycline loaded copper nanoparticles presented antibacterial activity against *Pseudomonas aeruginosa* and *Escherichia coli* (Yaqub et al., 2020); doxycycline complexed with silver nanoparticles presented antitumoral activity in an *in vitro* assay with MCF-7 breast cancer cell line (Mohammadkarimi et al., 2021).
Metal oxide nanoparticles	Iron oxide nanoparticles loaded with doxycycline presented extended drug release (Ghoderao et al., 2019); Zinc oxide nanoparticles loaded with doxycycline showed high drug loading capacity and antibacterial activity by UV photocatalysis (Suárez et al., 2017);
MSNs	Doxycycline incorporated in mesoporous nanosilica presented enhanced antimicrobial activity (Labban et al., 2021); chitosan patches containing MNSs with doxycycline improved skin wound healing in a rat model (Khosravian et al., 2022).
Carbon-based nanoparticles	Iron oxide-Carbon dots hybrid nanoparticles efficiently detected and degraded doxycycline in aqueous media (Kaur et al., 2022); nitrogen-doped carbon quantum dots efficiently identified doxycycline in pharmaceutical waste and bacterial cells (Raut et al., 2022)

SLNs = Solid lipid nanoparticles; NLCs = Nanostructured lipid carriers; PLGA = Poly(lactic-co-glycolic acid) (PLGA) nanoparticles; PEG = Polyethylene glycol (PEG) nanoparticles; MSNs = Mesoporous silica nanoparticles.

In CNS diseases, nanoparticles enhance drug delivery to the brain by crossing the BBB. They can mimic biological molecules like proteins, facilitating specific interactions with cells and tissues. Nanoparticles also shield drugs from degradation by the immune system, boosting therapeutic effectiveness and reducing off-target effects. Moreover, they can target specific CNS cells or tissues by modifying their surface with ligands that bind to specific cellular structures (Di Filippo et al., 2022; Teleanu et al., 2019; Joudeh and Linke, 2022).

Table 1 summarizes research on various types of nanoparticles and their role in AD treatment and DOX encapsulation. Nano-based drug delivery systems have generally shown promising results in both lab and animal studies. However, some nanoparticles, like nanostructured lipid carriers (NLCs) and polyethylene glycol-poly(lactic-co-glycolic acid (PEG-PLGA), lack sufficient research to gauge their effectiveness fully. Although NLCs are relatively new, studies already demonstrate their potential in AD and DOX delivery (Naseri et al., 2015; Sadegh et al., 2019; Shehata et al., 2023). PEG and PLGA are commonly combined to create nanoparticles, aiding drug delivery through different routes, such as intranasal and intravenous administration (Gao et al., 2006; Wani et al., 2020). While DOX is primarily known for its antimicrobial activity, ongoing research explores its efficacy in diverse fields, including neurodegenerative diseases, paving the way for optimized treatment strategies.

## 5 Conclusion

Doxycycline's repurposing for neurodegenerative diseases shows promise, with ongoing research focusing on its anti-amyloid, cholinergic, and neuroprotective effects. As a repurposed drug, the safety profile of DOX is well-established. What remains is to understand its effects on the brain when administered to the central nervous system. It is crucial to determine the appropriate doses to avoid poisoning and adverse effects. Additionally, the routes of administration targeting the brain need further exploration. For instance, the intranasal route may offer advantages over the intraperitoneal route, especially considering how easily the drug can cross the blood-brain barrier (BBB). Efforts are also directed towards optimizing its administration and delivery through nanostructured systems via in silico, *in vitro*, and *in vivo* studies to enhance understanding of DOX behavior on nanoparticles. This combined approach holds potential for advancing treatments for Alzheimer's and other brain-related conditions.

## CRediT authorship contribution statement

**Mariana Conceição:** Writing – review & editing, Writing – original draft, Formal analysis, Data curation. **Leonardo Delello Di Filippo:** Writing – original draft, Conceptualization. **Jonatas Lobato Duarte:** Writing – original draft, Conceptualization. **Fernando Pereira Beserra:** Writing – review & editing. **Maria Palmira Daflon Gremião:** Supervision. **Marlus Chorilli:** Writing – review & editing, Supervision, Conceptualization.

## Funding

This work was supported by São Paulo Research Foundation and 10.13039/501100002322Coordination for the Improvement of Higher Education Personnel (Grant numbers 2023/04282-2 and 001 88887.801886/2023-00).

We thank Kitty Jean Brown for reviewing the English writing of this work.

## Declaration of competing interest

There are no conflict of interest among all authors.

## Data Availability

No data was used for the research described in the article.
